# The role of FilGAP, a Rac‐specific Rho‐GTPase‐activating protein, in tumor progression and behavior of astrocytomas

**DOI:** 10.1002/cam4.937

**Published:** 2016-10-27

**Authors:** Atsuko Hara, Miki Hashimura, Koji Tsutsumi, Masashi Akiya, Madoka Inukai, Yasutaka Ohta, Makoto Saegusa

**Affiliations:** ^1^ Department of PathologyKitasato University School of Medicine1‐15‐1 Kitasato, Minami‐kuSagamiharaKanagawa252‐0374Japan; ^2^ Division of Cell BiologyDepartment of BiosciencesKitasato University School of Science1‐15‐1 Kitasato, Minami‐kuSagamiharaKanagawa252‐0374Japan

**Keywords:** Astrocytoma, cell morphology, FilGAP, IDH1, prognosis, Rac

## Abstract

FilGAP, a Rac‐specific Rho‐GTPase‐activating protein (GAP), acts as a mediator of Rho/ROCK‐dependent amoeboid movement, and its knockdown results in Rac‐driven mesenchymal morphology. Herein, we focused on the possible roles of FilGAP expression in astrocytomas. In clinical samples, FilGAP expression was significantly increased in grade (G) II astrocytomas as compared to normal astrocytes, but its expression strongly decreased in a grade‐dependent manner, and was positively associated with *isocitrate dehydrogenase 1 (IDH1)* mutations and inversely to cytoplasmic Rac1. Patients with astrocytoma showing a high FilGAP score had favorable overall survival as compared to the low score patients. Multivariate Cox regression analysis also showed that a high FilGAP score was a significant and independent favorable prognostic factor. Moreover, patients with high FilGAP score and *IDH1* mutant‐type astrocytomas had significantly the best Overall survival (OS) and Progression‐free survival (PFS), in contrast to the patients with low FilGAP score and wild‐type *IDH1* tumors who had the worst prognosis. In GIV tumors (GBM: glioblastomas), elongated tumor cells with low FilGAP expression were frequently observed in tumor core lesions, whereas the rounded cells with abundant expression were found in the peripheral areas adjacent to non‐neoplastic brain tissues. In an astrocytoma cell line, suppression of endogenous FilGAP expression by siRNAs caused an increased proportion of mesenchymal elongated cells, probably through increased Rac1 activity. These findings suggest that FilGAP, as well as IDH1 status, may be useful for predicting the behavior of astrocytomas. In addition, the FilGAP/Rac1 axis may serve as an important regulator of tumor progression in GBMs, probably through alteration of cell morphology.

## Introduction

Astrocytoma is the most common primary brain tumor and is subcategorized into World Health Organization (WHO) grade (G) II, GIII, and GIV [also referred to glioblastoma (GBM)] tumors characterized by astrocytic morphology with induced angiogenesis and/or necrosis 1. Recently, the *isocitrate dehydrogenase (IDH) 1* or *2* gene was found to be mutated in 50–80% of GII and GIII astrocytomas, and secondary GBMs 2. The most frequent type of *IDH1* mutation is G395A, which causes the amino acid substitution of arginine for histidine (R132H), whereas *IDH2* mutations are comparatively rare 3. In addition, *IDH1* mutations are considered to independently predict longer survival for patients with all grades of astrocytomas 4.

To infiltrate healthy brain tissue, astrocytoma cells must move through various tissues and cross tissue boundaries which require cell motility, remodeling of cell–cell contacts, and interaction with the extracellular matrix 5. Recently, two different modes of tumor cell movement have been proposed; the mesenchymal mode which is characterized by an elongated morphology and the amoeboid mode in which cells have a rounded morphology with no obvious polarity 6, 7, 8, 9, 10, 11, 12. Moving cells, particularly tumor cells, reciprocally switch between the two modes during cell migration 7.

Members of the Rho GTPase family, including RhoA, Rac, and Cdc42, are key regulators of cell migration by modulating mesenchymal and amoeboid motility 13, 14, 15, 16, 17. Rac is required for the formation of actin‐rich membrane ruffles, called lamellipodia, at the leading edge of the migrating cells, whereas RhoA regulates the formation of contractile actin‐myosin filaments, which form stress fibers, and maintains focal adhesion at the rear of the cells 14, 15, 16, 17. The Rho GTPases cycle between an inactive GDP‐bound form and active GTP‐bound form. The conversion to active status is catalyzed by guanine nucleotide exchange factors (GEF), and the return to the inactive state is by GTP‐activating proteins (GAP) 18.

FilGAP is a Rac‐specific Rho‐GAP and binds to the actin filament cross‐linking protein filamin A (FLNa) 6, 7, 19, 20. Knockdown of endogenous FilGAP induces a Rac‐driven elongated mesenchymal morphology, whereas its overexpression results in membrane blebbing and a rounded amoeboid morphology 6. Integrin *β* is also a filamin‐binding protein, and mechanical strain causes FilGAP to dissociate from FLNa/integrin *β* 21. In contrast, endothelial cell transforming factor (ECT) 2 is a Rac‐specific GEF, and its inhibition leads to decreased Rac1 activity with no change of Rho activity 22. However, little is known about the functional role of FilGAP in astrocytomas 23. In this study, we investigated the expression of FilGAP, with reference to the status of its related molecules, including FLNa, integrin *β*2, and Rac1, as well as the expression of ECT 2, cell proliferation, and *IDH1* gene status in astrocytomas. In addition, we examined whether FilGAP, as well as *IDH1* mutations, are suitable as prognostic factors and indicators of progression of astrocytomas.

## Materials and Methods

### Clinical cases

A total of 134 cases of astrocytomas, surgically resected at the Kitasato University hospital in the period from 1995 to 2013, were selected from our patient records according to the criteria of the 2007 WHO classification 1. The mean age of the patients was 48.5 years (range, 1–79 years). Of these, 53, 31, and 50 cases were subcategorized as GII, GIII, and GIV, respectively. None of the patients were treated with chemo‐radiation therapy before surgical resection of the tumors. In 38 GIV cases, tumor areas were subdivided into two categories, including tumor core and peripheral lesions adjacent to non‐neoplastic brain tissues. In addition, 18 samples of normal brain tissues around the tumors were applied. All tissues were routinely fixed in 10% formalin and processed for embedment in paraffin wax (FFPE). Approval for this study was given by the Ethics Committee of the Kitasato University School of Medicine (B14‐06).

### Antibodies

Rabbit polyclonal anti‐FilGAP antibody was developed as described previously 17, 19. Both anti‐FLNa and anti‐integrin *β*2 antibodies were purchased from Millipore (Billerica, MA). Anti‐ECT2 and anti‐Rac1 antibodies were bought from Santa Cruz Biotechnology (Santa Cruz, CA) and BD Bioscience (San Jose, CA), respectively. Anti‐IDH1 R132H antibody was obtained from Dianova GmbH (Hamburg, Germany). Anti‐*α*‐tubulin antibody was from Sigma‐Aldrich Chemicals (St. Louis, MO).

### Immunohistochemistry

Immunohistochemistry (IHC) was performed using a combination of microwave‐oven heating and polymer immunocomplex (Envision, Dako, Glostrup, Denmark) methods. The immunoreactions were visualized with DAB (3,3′ diaminobenzidine), and the nuclei were counterstained with methyl green.

For evaluation of the IHC findings, scoring for FilGAP, FLNa, integrin *β*2, cytoplasmic ECT2, and Rac 1 were carried out. Briefly, cases were subdivided into five categories on the basis of the proportion of immunopositive cells, as follows: 0, all negative; 1, <25%; 2, 25–50%; 3, 50–75%; and 4, >75% positive cells. The immunointensity was also subcategorized into four groups, as follows: 0, negative; 1, weak; 2, moderate; and 3, strong immunointensity. The IHC scores for each case were produced by multiplication of the values for the two parameters. To determine labeling indices (LIs) for nuclear ECT2 and Ki‐67 immunoreactivity, the immunopositive nuclei of at least 700 tumor cells were counted in five randomly selected fields. The LIs were then calculated as number per 100 cells. In GBM cases, the IHC score and nuclear LI were also examined in tumor core and peripheral lesions separately. Immunopositivity for IDH1‐R132H was considered when a large proportion of tumor cells showed strong cytoplasmic immunoreaction as described previously 24. In addition, cases were defined as positive for plasma membrane and/or perinuclear Rac1 immunoreactivity when over 10% of the cells were stained in each section.

### *In situ* hybridization

Riboprobes for FilGAP containing nucleotides 1027 to1726 of the *FilGAP* gene were generated by in vitro transcription using full length FilGAP cDNA 10, and *In situ* hybridization (ISH) assays were performed using the GenPoint Tyramide Signal Amplification System (Dako), as described previously 25. Cases with more than 10% cells positive for ISH signals were defined as positive.

### DNA extraction, polymerase chain reaction (PCR), and direct DNA sequencing

Genomic DNA was extracted from FFPE sections using the QIAamp DNA FFPE Tissue Kit (Qiagen, Valencia, CA), according to the manufacturer's instructions. Exon 4 of the *IDH1* gene was amplified and sequenced as described previously 26.

### Cell culture, siRNA, and transfection

Five human glioma cell lines, U87MG [GBM, American Type Culture Collection (ATCC), Manassas, VA], KS‐1 [GBM, Health Science Research Resources Bank (HSRRB), Osaka, Japan], KINGS‐1 (GIII astrocytoma, HSRRB), no.10 (GIII astrocytoma, HSRRB), and SF‐126 (GBM, HSRRB) cell lines were maintained in DMEM, Eagle's MEM, or RPMI1640 with 10% bovine calf serum.

siRNA oligonucleotide duplexes targeting human *FilGAP* were purchased from Invitrogen (Carlsbad, CA). KINGS‐1 cells were transfected with control or FilGAP siRNA using Lipofectamine RNAi max reagent (Invitrogen) and cultured on plastic plates for 48 h. The control or transfected cells were trypsinized, plated on top of a thick deformable layer of type I collagen, and cultured for 24 h. For visualization of F‐actin, cells were stained with Alexa Fluor 568‐phalloidin as described previously 6. At least 500 cells were counted to determine those with elongated or rounded morphology, and the LIs were then calculated as average number per 100 cells from three independent experiments.

### Western blot assays

Total cellular proteins were harvested using RIPA buffer [20 mmol/L Tris‐HCl (pH7.2), 1% Nonidet P‐40, 0.5% sodium deoxycholate, and 0.1% sodium dodecyl sulfate]. Aliquots of the proteins were resolved by SDS‐PAGE, transferred to membranes, and probed with primary antibodies, and the signals were visualized with the ECL detection system (Amersham Pharmacia Biotechnology, Tokyo, Japan).

### Rho GAP assay

Cell lysates extracted by RIPA buffer were precleared, and the supernatant was assessed to determine the amount of total Rac protein, and the remaining fluid was incubated with 20 *μ*g of GST‐PAK‐CRIB protein in the presence of glutathione–Sepharose beads. The beads were washed and the level of GTP‐bound Rac1 was determined by western blot assay using an anti‐Rac1 antibody.

### Cell proliferation assay

KINGS‐1 cells were transfected with FilGAP siRNA by Lipofectamine RNAi max reagent (Invitrogen) according to the manufacturer's reverse transfection protocol. After 48 h, transfected cells were trypsinized and 1 × 10^4^, 2 × 10^4^, or 5 × 10^4^ cells were seeded on 96‐well plates with or without 1.7 mg/mL rat tail collagen. Cells were cultured for 6 days, and cell proliferation was measured using an MTT‐based cell proliferation kit (Roche, Tokyo, Japan) according to the manufacturer's protocol.

### Statistics

Comparative data were analyzed using the Tukey's test, chi‐square test, and the Spearman's correlation coefficients. Overall survival (OS) was calculated as the time between onset and death or the date of the last follow‐up evaluation. Progression‐free survival (PFS) was also examined from the onset of treatment until relapse, disease progression, or last follow‐up evaluation. OS and PSF were estimated using the Kaplan–Meier method, and the statistical comparisons were made using the log rank test. Univariate and multivariate analyses were performed using the Cox proportional hazards regression model. The cut‐off for statistical significance was set as *P* < 0.05.

## Results

### Expression of FilGAP and its associated molecules in astrocytomas

In normal brain tissues, immunoreactivity for FilGAP, FLNa, integrin *β*2, ECT2, Rac1, and Ki‐67 was extremely low or absent in normal astrocytes, as well as nerve cells (Fig. S1).

**Figure 1:**
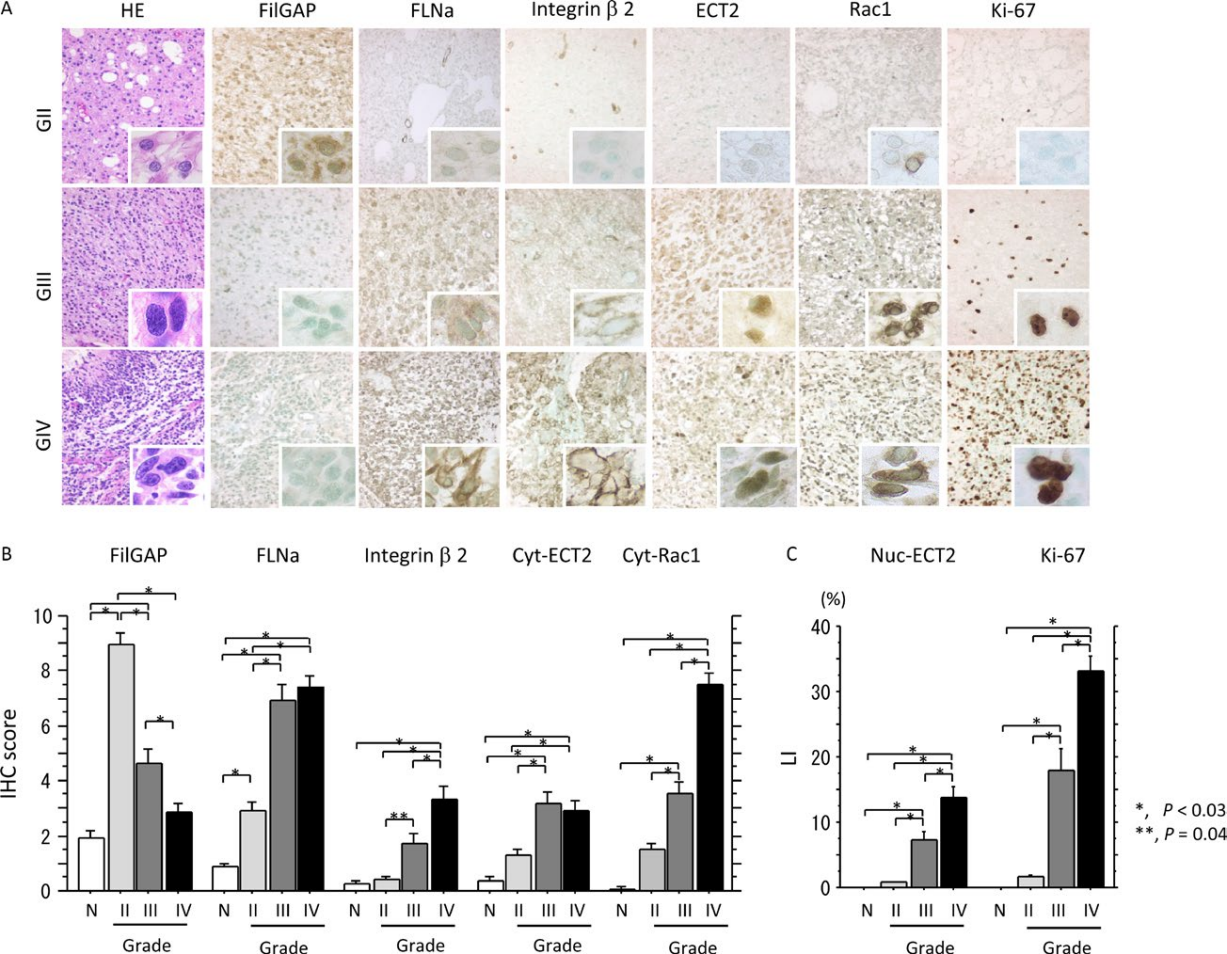
Expression of FilGAP and its related molecules in astrocytomas. (A) Staining by hematoxylin and eosin (HE) and by Immunohistochemistry (IHC) for FilGAP, FLNa, integrin *β*2, endothelial cell transforming factor (ECT)2, Rac1, and Ki‐67 in grade (G) II, III, and IV tumors. Some astrocytoma cells are magnified in the insets. Original magnification, ×100 and ×400 (inset). (B) IHC scores for FilGAP, FLNa, integrin *β*2, and cytoplasmic (Cyt) ECT2 and Rac1, and (C) labeling indices (LIs) of nuclear (Nuc) ECT2 and Ki‐67 in normal astrocytes (N) and grade II, III, and IV astrocytomas. The data shown are means±SDs.

Representative IHC findings for FilGAP, FLNa, integrin *β*2, ECT2, Rac1, and Ki‐67 in astrocytomas are illustrated in Figure 1A. Immunoreaction for FilGAP was mainly observed in cytoplasmic and/or nuclear compartments of astrocytoma cells. In 17 astrocytoma cases, FilGAP immunoreactivity was positively correlated (*ρ *= 0.87, *P* = 0.005) to its mRNA signals as detected by ISH assay (Fig. S2). Immunoreactivity for Rac1, FLNa, integrin *β*2, and Ki‐67 was located in either the nuclear or cytoplasmic component. Immunoreactivity for Rac1 in the plasma membrane and/or perinuclear region was also observed in astrocytoma cells with relatively intense cytoplasmic staining and was found to have significant positive association with cytoplasmic Rac1 (Fig. S3). In addition, its immunopositivity showed a significant stepwise increase from GII through GIII to GIV tumors (Table S2).

Average FilGAP score was significantly higher in GII tumors as compared to normal astrocytes and showed significant stepwise decrease from GII through GIII to GIV tumors. In contrast, IHC scores for FLNa, integrin *β*2, cytoplasmic ECT2, and cytoplasmic Rac1, as well as LI values for nuclear ECT2 and Ki‐67, were significantly increased from normal astrocytes to GIV lesions in a grade‐dependent manner (Fig. 1B).

Overall, FilGAP scores were inversely correlated with cytoplasmic Rac1 scores and LIs of nuclear ECT2 and Ki‐67. In contrast, cytoplasmic Rac1 scores were positively correlated with FLNa and integrin *β*2 scores and LIs of nuclear ECT2 and Ki‐67. There was also a positive correlation between FLNa and integrin *β*2 scores (Table 1).

**Table 1:** Correlations among expression of FilGAP and its related molecules in all grades of astrocytomas

	FilGAP	Filamin A	Integrin *β*2	Cyt‐ECT2	Nuc‐ECT2	Cyt‐Rac1
	*ρ* (*P*)	*ρ* (*P*)	*ρ* (*P*)	*ρ* (*P*)	*ρ* (*P*)	*ρ* (*P*)
Filamin A	−0.32 (0.0002)	*	*	*	*	*
Integrin *β*2	−0.25 (0.04)	0.45 (<0.0001)	*	*	*	*
Cyt‐ECT2	−0.18 (0.04)	0.36 (<0.0001)	0.32 (0.0003)	*	*	*
Nuc‐ECT2	−0.52 (<0.0001)	0.35 (<0.0001)	0.27 (0.002)	0.16 (0.33)	*	*
Cyt‐Rac1	−0.47 (<0.0001)	0.52 (<0.0001)	0.51 (<0.0001)	0.24 (0.008)	0.46 (<0.0001)	*
Ki‐67	−0.57 (<0.0001)	0.26 (0.003)	0.31 (0.0004)	0.31 (0.0004)	0.75 (<0.0001)	0.49 (<0.0001)

*ρ*, Speraman's correlation coefficient; *, not examined; Cyt, cytoplasmic; Nuc, nuclear; ECT, endothelial cell transforming factor.

### Association of IDH1 gene status with FilGAP in astrocytomas

**Figure 2:**
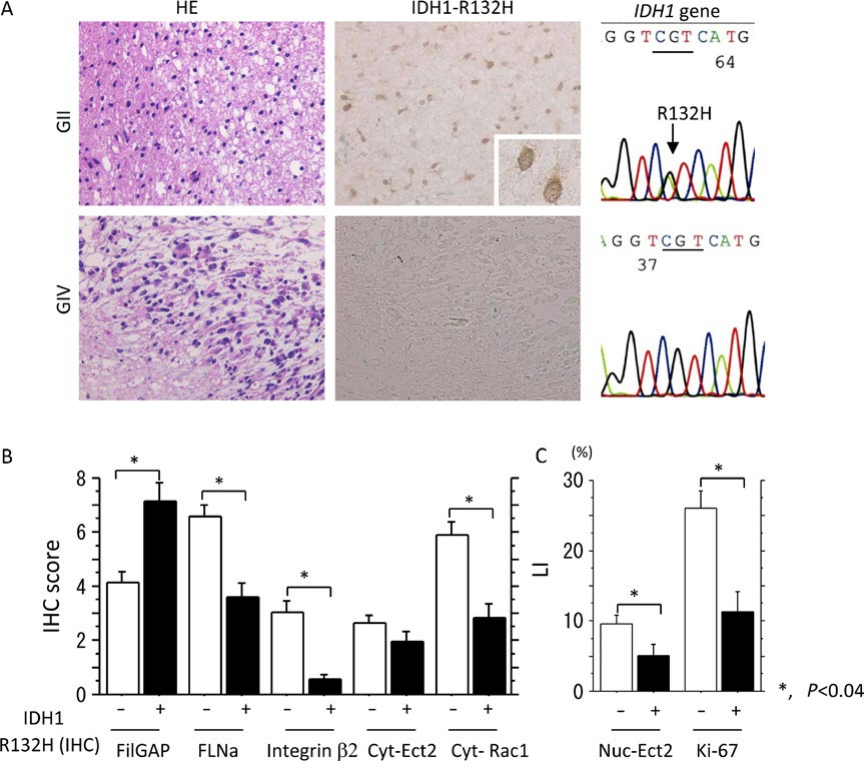
Isocitrate dehydrogenase 1 (IDH1) abnormality and its associations with FilGAP and its related molecules. (A) Immunohistochemistry (IHC) and sequence analysis of IDH1 in grade (G) II and GIV astrocytomas. Note the cytoplasmic IDH1 staining (middle) and heterozygous mutation (R132H) of *IDH1* gene (right) in GII astrocytoma (upper), in contrast to a lack of such findings in GIV tumor (lower). (B) IHC scores for FilGAP, FLNa, integrin *β*2, and cytoplasmic (Cyt) endothelial cell transforming factor (ECT)2 and Rac1, and (C) labeling indices (LIs) of nuclear (Nuc) ECT2 and Ki‐67 between IDH1R132H‐positive and ‐negative astrocytomas. The data shown are means±SDs.

Representative IHC and mutational analysis for the *IDH1* gene in GII and GIV astrocytomas are shown in Figure 2A. Strong immunoreactivity for IDH1‐R132H mutant protein was mainly observed in cytoplasmic areas and was positively correlated with the gene mutations affecting codon 132 (CGT to CAT) (Table S1). IDH1 alterations were significantly detected in GII and GIII astrocytomas as compared to GIV tumors (Table 2). In addition, immunopositivity for the IDH‐R132H mutant protein was positively associated with FilGAP score and negatively to other markers (Fig. 2B). Similar associations of the gene mutation with IHC score and LIs were also observed, with the exception of FilGAP score (*P* = 0.06) and Ki‐67 LI (*P* = 0.06) (Fig. S4).

**Table 2:**
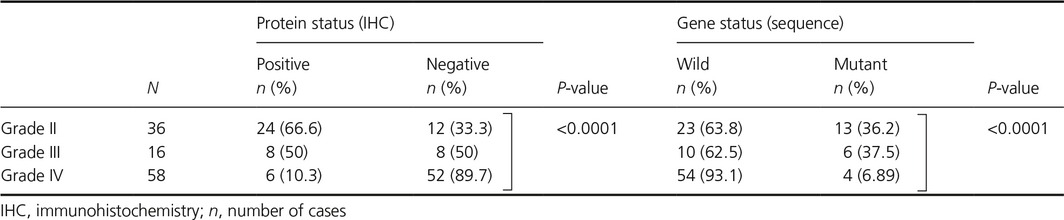
Alteration in isocitrate dehydrogenase 1 status in astrocytomas

### Association of expression of FilGAP and its related molecules with prognosis in astrocytomas

To evaluate the prognostic significance of expression of FilGAP and its related molecules, as well as IDH1 status, the scores or LIs were divided into two categories (high and low) with the mean values as the cut‐off in each category (Tables 3 and 4).

**Table 3:** Univariate analysis for overall survival and progression‐free survival in astrocytoma patients

Variables	Cut‐off	GII+GIII+GIV		Cut‐off	GII+GIII		Cut‐off	GIV		Favorable factor
	Low/High	Log rank c2	*P*‐value	Low/High	Log rank c2	*P*‐value	Low/High	Log rank c2	*P*‐value	
Overall survival (OS)										
FilGAP	5/6	10.14	0.001	8/9	4.48	0.03	3/4	1.18	0.28	High score
FLNa	6/7	3.08	0.08	3/4	1.44	0.23	8/9	0.1	0.75	*
Integrin *β*2	2/3	3.69	0.05	1/2	1.19	0.27	3/4	1.07	0.3	Low score
Cyt‐ECT2	3/4	0.03	0.87	1/2	1.46	0.23	3/4	0.07	0.79	*
Nuc‐ECT2	7/7.1	16.1	<0.0001	3/3.1	3.65	0.06	13/13.1	0.18	0.67	Low score
Cyt‐Rac1	5/6	5.75	0.01	2/3	0.17	0.68	7/8	1.52	0.22	Low score
Ki‐67	17/17.1	3.42	0.06	7/7.1	1.29	0.53	33/33.1	1.53	0.22	*
Age	49/50	12.72	0.0004	43/44	4.82	0.03	57/58	0.21	0.64	Young
IDH1 (IHC)	Neg/Posi	9.1	0.003	Neg/Posi	1.37	0.24	Neg/Posi	3.17	0.07	Positive
*IDH1* gene	Wild/Mut	6.03	0.014	Wild/Mut	2.31	0.13	Wild/Mut	2.09	0.15	Mutant
Progression‐free survival (PFS)										
FilGAP	5/6	12.97	0.0003	8/9	8.89	0.003	3/4	0.12	0.72	High score
FLNa	6/7	4.65	0.03	3/4	5.29	0.02	8/9	0.39	0.53	Low score
Integrin *β*2	2/3	7.62	0.006	1/2	0.55	0.46	3/4	0.005	0.94	Low score
Cyt‐ECT2	3/4	0.01	0.91	1/2	3.07	0.08	3/4	0.78	0.38	*
Nuc‐ECT2	7/7.1	34.63	<0.0001	3/3.1	13.21	0.0003	13/13.1	0.05	0.82	Low score
Cyt‐Rac1	5/6	18.6	<0.0001	2/3	0.2	0.65	7/8	1.16	0.28	Low score
Ki‐67	17/17.1	26.46	<0.0001	7/7.1	5.97	0.05	33/33.1	1.09	0.3	Low score
Age	49/50	19.23	<0.0001	43/44	7.74	0.005	57/58	0.97	0.32	Young
IDH1 (IHC)	Neg/Posi	7.34	0.007	Neg/Posi	0.06	0.81	Neg/Posi	4.01	0.04	Positive
*IDH1* gene	Wild/Mut	8.61	0.003	Wild/Mut	1.14	0.28	Wild/Mut	5.67	0.02	Mutant

G, grade; Hist, histological; Cyt, cytoplasmic; Nuc, nuclear; IHC, immunohistochemistry; Neg, negative; Posi, positive; Mut, mutant; ECT2, endothelial cell transforming factor 2; *IDH,* isocitrate dehydrogenase 1.

*Not significant.

**Table 4:** Multivariate analysis for overall survival and progression‐free survival in astrocytoma patients

Variables	Cut‐off	GII+GIII+GIV			Cut‐off	GII+GIII			Cut‐off	GIV		
	(Low/High)	Hazard Ratio	95% CI	*P*‐value	(Low/High)	Hazard Ratio	95% CI	*P*‐value	(Low/High)	Hazard Ratio	95% CI	*P*‐value
Overall survival (OS)												
FilGAP	5/6	−0.17	−0.35–0.001	0.05	8/9	−0.1	−2.14–1.65	0.91	3/4	−0.96	−2.08–−0.11	0.02
FLNa	6/7	−0.003	−0.22–0.2	0.97	3/4	−0.86	−3.47–0.47	0.39	8/9	−0.07	−0.92–0.71	0.85
Integrin *β*2	2/3	−0.14	−0.4–0.08	0.23	1/2	0.62	−1.04–2.07	0.41	3/4	−1.1	−2.3–0.11	0.03
Cyt‐ECT2	3/4	−0.08	−0.37–0.18	0.57	1/2	−0.57	−2.38–1.09	0.49	3/4	0.6	−0.18–1.38	0.13
Nuc‐ECT2	7/7.1	−0.02	−0.09–0.05	0.59	3/3.1	−0.94	−2.72–0.49	0.19	13/13.1	−0.17	−1.06–0.68	0.69
Cyt‐Rac1	5/6	0.01	−0.17–0.19	0.87	2/3	0.74	−1.07–2.95	0.42	7/8	−0.42	−1.19–0.31	0.25
Ki‐67	17/17.1	0.03	−0.02–0.07	0.24	7/7.1	1.07	−0.44–2.71	0.16	33/33.1	0.7	−0.07–1.62	0.08
Age	49/50	0.02	−0.01–0.06	0.21	43/44	1.4	0.33–2.87	0.008	57/58	0.22	−0.73–1.3	0.66
IDH1 (IHC)	Neg/Posi	−0.42	−2.01–0.98	0.56	Neg/Posi	0.09	−0.95–1.12	0.86	Neg/Posi	−11.54	−28044–28021	0.007
*IDH1* gene	Wild/Mut	−1.62	−4.67–0.44	0.13	Wild/Mut	−0.82	−2.38–0.32	0.16	Wild/Mut	−8.71	−35951–35934	0.82
Progression‐free survival (PFS)												
FilGAP	5/6	−0.11	−0.23–0.01	0.08	8/9	−1.61	−3.16–−0.39	0.009	3/4	0.04	−0.57–0.59	0.89
FLNa	6/7	−0.02	−0.15–0.11	0.68	3/4	−0.51	−0.32–0.98	0.45	8/9	−0.23	−0.31–0.015	0.36
Integrin *β*2	2/3	−0.008	−0.17–0.13	0.92	1/2	−9.73	−28379–28359	0.45	3/4	−0.4	−1.03–0.17	0.17
Cyt‐ECT2	3/4	−0.1	−0.3–0.08	0.27	1/2	1.49	0.08–3.37	0.04	3/4	0.07	−0.46–0.54	0.79
Nuc‐ECT2	7/7.1	−0.01	−0.06–0.04	0.71	3/3.1	−0.4	−2.29–1.65	0.67	13/13.1	0.2	−0.26–0.68	0.39
Cyt‐Rac1	5/6	0.08	−0.03–0.19	0.17	2/3	−0.34	−1.51–0.75	0.53	7/8	−0.16	−0.67–0.33	0.52
Ki‐67	17/17.1	0.03	−0.002–0.06	0.07	7/7.1	−0.02	−1.75–1.35	0.98	33/33.1	0.08	−0.43–0.61	0.75
Age	49/50	0.02	−0.004–0.04	0.09	43/44	−0.23	−1.53–0.96	0.7	57/58	0.17	−0.36–0.73	0.54
IDH1 (IHC)	Neg/Posi	0.62	−0.32–1.55	0.19	Neg/Posi	−0.38	−0.58–1.41	0.43	Neg/Posi	−0.59	−1.51–0.25	0.17
IDH1 gene	Wild/Mut	−1.17	−2.39–0.09	0.03	Wild/Mut	0	–	–	Wild/Mut	−0.72	−2.26–0.36	0.2

G, grade; Hist, histological; CI, Confidence Interval; Cyt, cytoplasmic; Nuc, nuclear; IHC, immunohistochemistry; Neg, negative; Posi, positive; Mut, mutant; IDH 1, isocitrate dehydrogenase 1; ECT2, endothelial cell transforming factor 2

**Figure 3:**
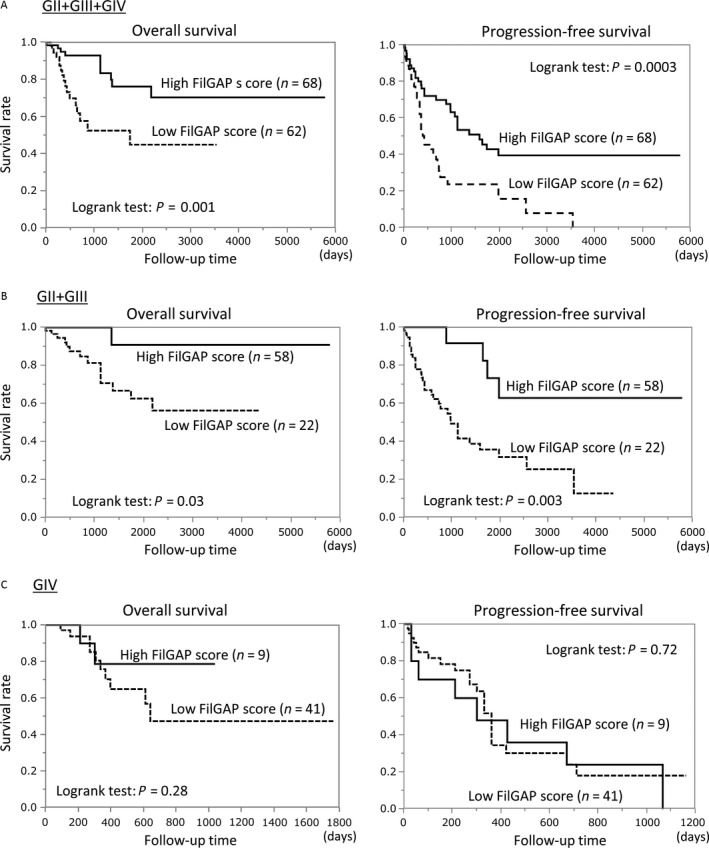
Relationship of FilGAP expression with overall survival (OS) and progression‐free survival (PFS) in astrocytomas. OS (left) and PFS (right) of astrocytoma patients with GII/GIII/GIV (A), GII/GIII (B), and GIV (C) astrocytomas. *N*, number of cases.

The Kaplan–Meier curves for OS and PFS with respect to FilGAP expression in astrocytomas showed that the patients with all grades of astrocytomas who displayed high FilGAP scores had more favorable OS and PFS as compared to the low FilGAP score patients (Fig. 3A). Similar associations were also observed in GII/GIII (Fig. 3B), but not GIV, astrocytomas (Fig. 3C).

Univariate Cox proportional hazards regression revealed that the FilGAP score as well as nuclear ECT2 LI, cytoplasmic Rac1 score, age, and IDH1 status were significant prognostic factors for OS in all grades of tumors. Further investigation showed that the FilGAP score and age appeared to have significant prognostic value for OS in GII/GIII, but not GIV, tumors. In addition, most of the factors we investigated also showed significant prognostic value for PFS in all grades of tumors. Further examination showed significant prognostic value of FilGAP and FLNa scores in GII/GIII tumors, and IDH1 status in GIV tumors for PSF (Table 3). Multivariate Cox regression analysis showed that FilGAP expression, as well as some markers, appeared to be a significant and independent prognostic factor for OS, but not PFS, in all including GIV tumors (Table 4).

**Figure 4:**
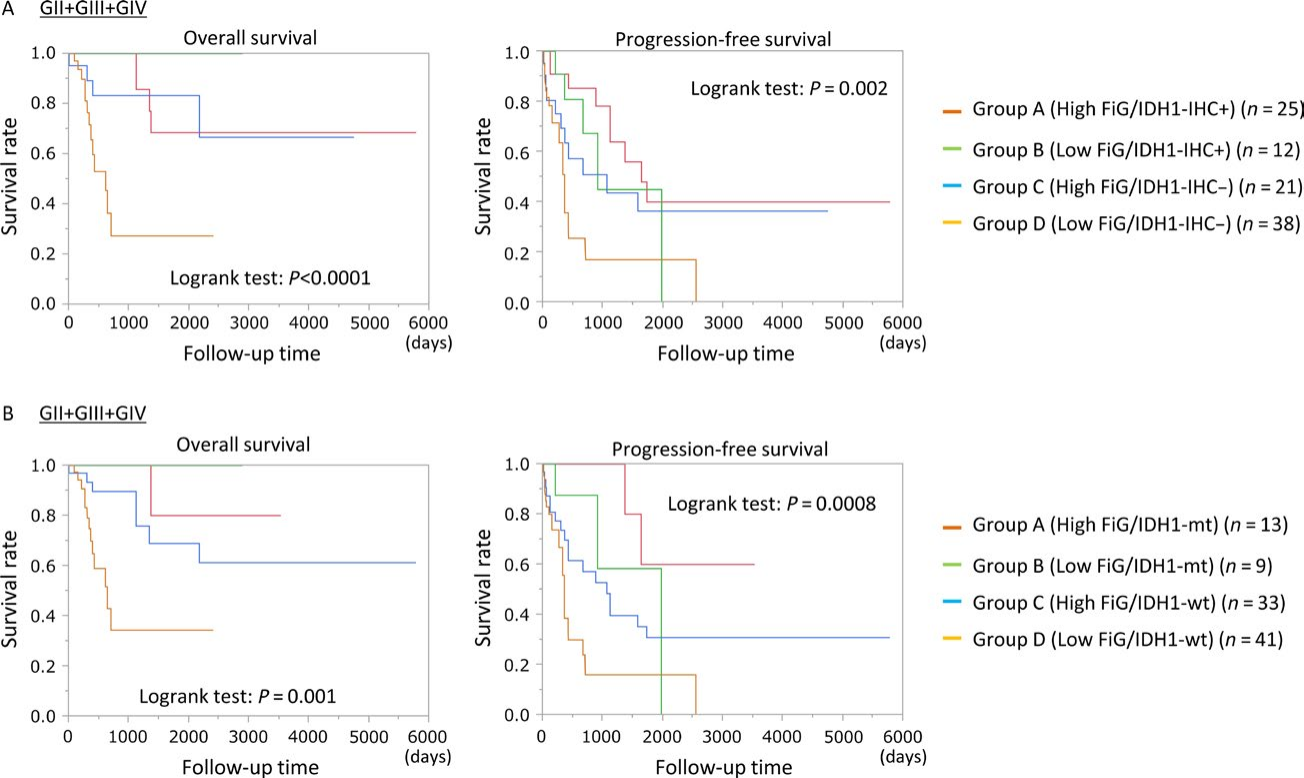
Relationship of FilGAP expression and Isocitrate dehydrogenase 1 (IDH1) status with overall survival (OS) and progression‐free survival (PFS) in astrocytomas. (A) OS (left) and PFS (right) among four groups stratified by combined FilGAP and IDH1R132H expression (A) or the gene mutation status (B) in GII/GIII/GIV astrocytomas. N, number of cases.

To further examine the prognostic significance of FilGAP in molecular classification of astrocytomas, the patients were subdivided into four groups, on the basis of FilGAP scores and IDH1 status. As shown in Figure 4, Group A (high FilGAP score/high IDH1 expression or mutation) appeared to have the best OS and PFS, whereas patients of Group D (low FilGAP score/no IDH1 expression or mutation) had the worst prognosis.

### Difference in FilGAP expression between tumor core and peripheral lesions in GBMs

**Figure 5:**
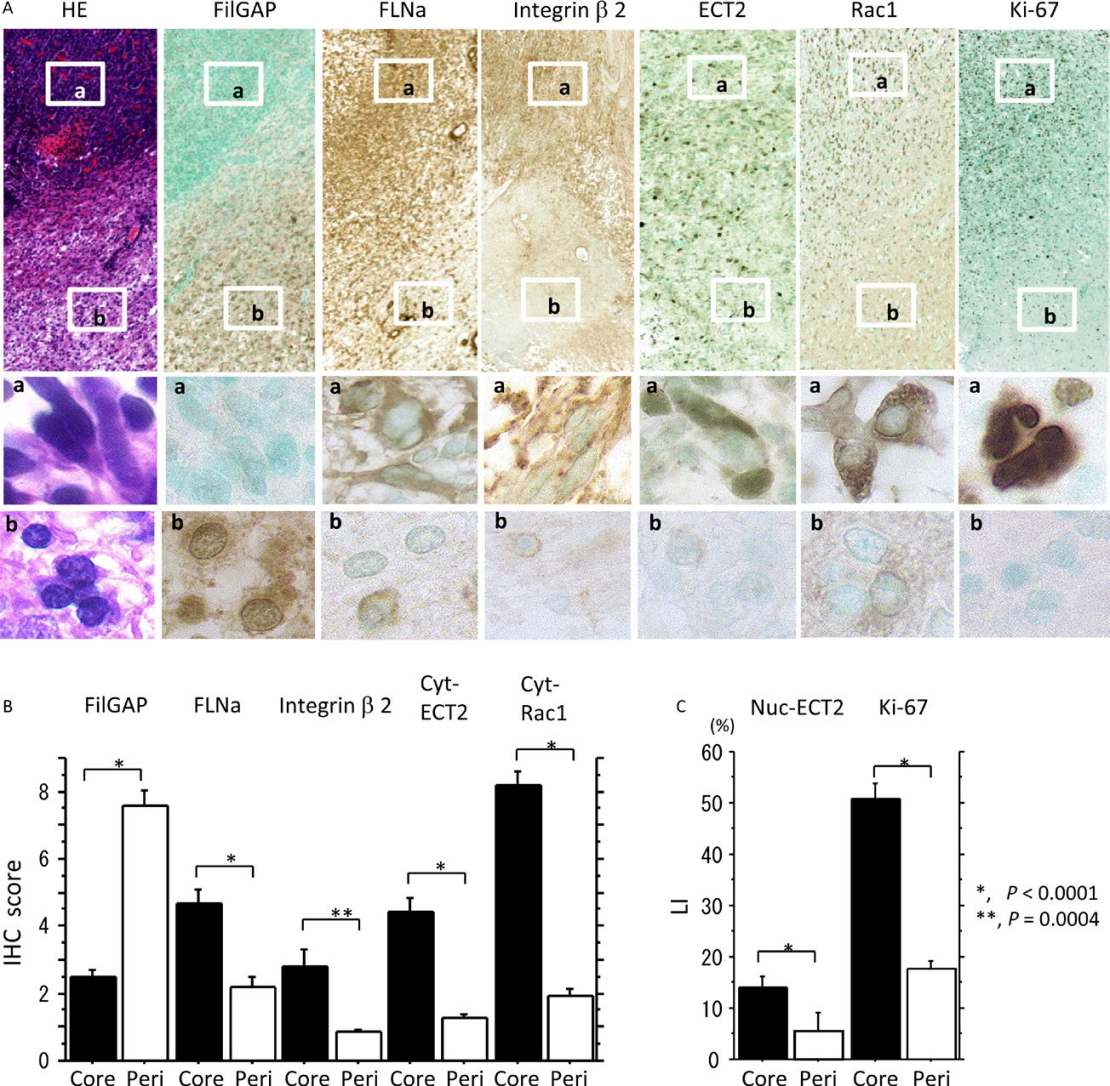
Expression of FilGAP and its related molecules in tumor core and peripheral lesions in glioblastoma (GBM). (A) Staining by hematoxylin and eosin (HE) and by Immunohistochemistry (IHC) for FilGAP, FLNa, integrin *β*2, endothelial cell transforming factor (ECT)2, Rac1, and Ki‐67. Boxes enclose a magnified view of the (a) core and (b) peripheral lesions. Note the elongated tumor cells in the core lesion and the rounded cells in the peripheral areas within GBM. Original magnification, ×100 and ×400 (inset). (B) IHC scores for FilGAP, FLNa, integrin *β*2, and cytoplasmic (Cyt) ECT2 and Rac1, and (C) labeling indices (LIs) of nuclear (Nuc) ECT2 and Ki‐67 in tumor core (Core) and peripheral (Peri) lesions in GBM. The data shown are means±SDs.

Since it is known that GBM shows extensive dissemination along white matter tracts with poorly defined infiltrative borders 27, we examined for differences in expression of FilGAP and its related molecules between the tumor core and the periphery adjacent to non‐neoplastic brain tissues in GBMs. Representative IHC findings for FilGAP, FLNa, integrin *β*2, ECT2, Rac1, and Ki‐67, along with typical morphological features of tumor cells in these areas, are illustrated in Figure 5A.

The average FilGAP score was significantly higher in the peripheral lesions as compared to the core lesions within tumor tissues, in contrast to significantly higher FLNa, integrin *β*2, cytoplasmic ECT2, and cytoplasmic Rac1 scores, as well as plasma membrane/perinuclear Rac1 immunopositivity, and nuclear ECT2 and Ki‐67 LIs in the latter (Fig. 5B and Table S2). The FilGAP score was inversely correlated with FLNa, cytoplasmic ECT2, and cytoplasmic Rac1 scores and Ki‐67 LIs. The cytoplasmic Rac1 score was positively correlated with FLNa, integrin *β*2, cytoplasmic ECT2 scores, and Ki‐67 LI. Positive correlations among the FLNa, cytoplasmic ECT2, and integrin *β*2 scores and nuclear ECT2 and Ki‐67 LIs were also found (Table S3).

### Relationship between FilGAP expression and amoeboid‐mesenchymal transition in astrocytoma cells

**Figure 6:**
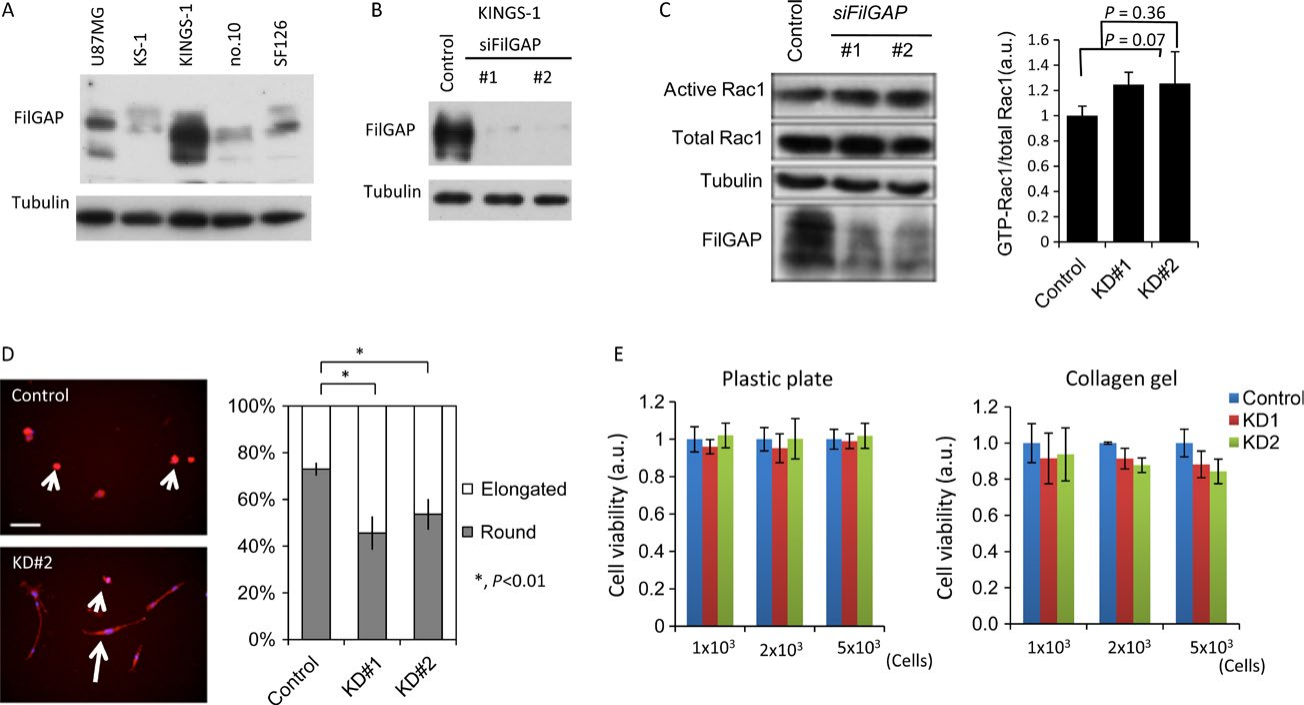
Relationship of FilGAP expression with cell morphology and proliferation in astrocytoma cells. (A) Western blot analysis for FilGAP expression in the indicated astrocytoma cell lines. *α*‐tubulin was used as an internal loading control. (B) Inhibition of FilGAP expression by transfection with specific siRNAs (#1 and #2) in KINGS‐1 cells. (C) Left: KINGS‐1 cells were transfected with control or FilGAP siRNAs for 2 days. Cell extracts were prepared and incubated with GST‐PAK‐CRIB protein that was immobilized on glutathione‐Sepharose beads. The amount of Rac1 in cell lysates before pull‐down and GTP (GST‐PAK‐CRIB‐bound) Rac1 was detected by western blot assay. Expression levels of FilGAP and *α*‐tubulin (loading control) are also shown. Right: the relative expression of GTP‐Rac to its total form. The data shown are means±SDs. The experiment was performed in sextuplicate. (D) Left: representative images of KINGS‐1 cells with rounded (indicated by short arrows) and elongated (indicated by long arrow) morphology. Scale bar: 100 *μ*m. Right: the proportion of KINGS‐1 cells with rounded and elongated morphology following knockdown of FilGAP expression (KD#1 and KD#2). The data shown are means±SDs. The experiment was performed in triplicate. (E) Cell viability of KINGS‐1 cells plated on plastic plate (upper) and collagen gel (lower) following knockdown of FilGAP expression (KD#1 and KD#2). The data shown are means±SDs.

To examine the role of FilGAP in cell morphology, we selected KINGS‐1 cells, which have abundant endogenous FilGAP expression, from among five astrocytoma cell lines (Fig. 6A). Two independent siRNAs targeting FilGAP (KD#1 and KD#2) dramatically reduced the expression of endogenous FilGAP (Fig. 6B), resulting in slightly increased expression of GTP‐Rac1 as compared to that of its total form (Fig. 6C) and a significant increase in the proportion of mesenchymal‐type elongated cells in a three‐dimensional environment (Fig. 6D). However, the suppression did not have any effect on cell proliferation in KINGS‐1 cells under both plastic and collagen gel conditions (Fig. 6E).

## Discussion

This study clearly provided evidence that cytoplasmic Rac1 immunoreactivity showed a strong grade‐dependent increase in astrocytomas. Plasma membrane/perinuclear staining appeared to be also relatively common in tumor cells with intense cytoplasmic staining. Rac1 is posttranscriptionally regulated, either by an increase in RNA stability, translation efficiency, and/or protein stability in GBMs 28. In addition, inactivated Rac1 resides in the cytoplasm, whereas activated Rac1 forms relocalize to the plasma membrane and/or perinuclear vesicles 29, 30. Given our results of a significantly positive association between plasma membrane/perinuclear and cytoplasmic Rac1 expression, abundant cytoplasmic Rac1 expression, as well as its plasma membrane and/or perinuclear immunoreactivity, appeared to be required for an increase in its active form in astrocytomas, in line with increased malignant potential. In fact, the OS and PFS of patients with high Rac1 scores appeared to be significantly poorer than those with Rac1‐negative tumors, again indicating that Rac1 activity may contribute to the aggressive feature of astrocytomas.

ECT2 is a GEF that is typically expressed in the nucleus during interphase until mitosis and is exported from the nucleus into the cytoplasm where it activates RhoA to regulate assembly of the actomyosin contractile ring during metaphase 22, 26, 31. From our results, although both nuclear and cytoplasmic ECT2 expression showed significant grade‐dependent increases in astrocytomas, the nuclear, but not cytoplasmic, ECT2 scores were positively correlated with cytoplasmic Rac1 expression. In addition, the low nuclear rather than cytoplasmic levels of ECT2 appeared to be a favorable prognostic factor for OS and PFS in astrocytomas, although the nuclear form is thought to reflect the GEF in its inactive state 32. At the present time, although we are unable to provide an appropriate explanation for the observations, it appears that the functional role of ECT2 in Rac1 signaling may be very complex and dependent on some cell type‐specific factors.

In this study, FilGAP expression was significantly increased in GII astrocytomas as compared to normal astrocytes, but its expression was strongly decreased in a grade‐dependent manner. Further, it was inversely correlated with cytoplasmic Rac1 expression as well as that of FLNa and nuclear ECT2. In general, an interaction between FLNa and FilGAP appears to be a key factor in maintaining low levels of active Rac in mechanically challenged cells 33. Moreover, mechanical strain increases *β*‐integrin binding to FLNa, whereas it causes FilGAP to dissociate from FLNa 21. Given our results that showed positive correlations among cytoplasmic Rac1, FLNa, and integrin *β*2, it is likely that FilGAP may serve as a key regulator for modulation of Rac1 activity through its interaction with the FLNa/integrin *β*2 axis in astrocytoma cells.

Importantly, the immunohistochemical analysis of FilGAP expression was able to delineate its relationship with prognosis in astrocytomas. The OS and PFS of patients showing high FilGAP scores were significantly more favorable than those with low FilGAP expression according to the Kaplan–Meier survival curves. Moreover, FilGAP expression was an independent prognostic factor in all including GIV categories of astrocytomas as shown by Cox regression analysis. In addition, the patients with high FilGAP score and *IDH1*‐mt tumors had the best OS and PFS, in contrast to the patients with low FilGAP score and wild‐type (wt) *IDH1* astrocytomas who had the worst prognosis. These findings suggest that FilGAP‐positive astrocytomas may constitute a unique subtype with a favorable clinical course and a combination of FilGAP and IDH1 profiles has potential importance for predicting the prognosis.

Based on the findings that demonstrated an inverse correlation between the FilGAP score and cell proliferation in astrocytoma tissues, inhibition of cell proliferation in response to a high FilGAP expression was expected. However, silencing of endogenous FilGAP by specific siRNAs did not have any effect on cell proliferation in KINGS‐1 cells, suggesting that the suppressive effects of FilGAP on Rac1 activity may be an end result of decreased cell proliferation through stimulation of various signal pathways such as those that control cell cycle progression.

**Figure 7:**
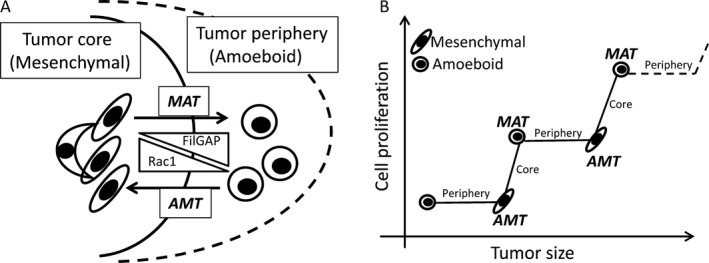
Schematic representation of the FilGAP/Rac1 axis during tumor progression and invasion in GBMs. (A) Association of FilGAP and Rac1 expression with mesenchymal‐amoeboid and amoeboid‐mesenchymal transitions (MAT and AMT) in tumor core and peripheral lesions in astrocytoma. (B) Reciprocal switch between AMT and MAT during tumor progression of GBM cells.

Another interesting finding in this study was that elongated and fibroblast‐like tumor cells lacking FilGAP expression were frequently observed in tumor cores in GBMs, whereas rounded cells with abundant expression were found in the peripheral areas. In addition, these cells demonstrated inverse correlations of FilGAP expression with cytoplasmic Rac1 as well as FLNa, integrin *β*2, and cytoplasmic ECT2. In KINGS‐1 cells, suppression of endogenous FilGAP expression by specific siRNAs resulted in an increased proportion of mesenchymal‐type elongated features. These findings appear to be in line with the evidence that high Rac activity by suppression of FilGAP expression promotes amoeboid‐mesenchymal transition (AMT), whereas its forced expression induces the opposite effects mesenchymal‐amoeboid transitions (MAT) 6, 34. In fact, plasma membrane/perinuclear Rac1 immunopositivity, which is closely linked to its active form 29, 30, was significantly lower in peripheral tumor lesions as compared to the core areas of GBMs. Given that the mesenchymal mode of motility requires extracellular proteolysis, whereas the amoeboid mode is independent of proteases 5, the two interconvertible modes may participate in tumor progression and invasion in GBMs through the FilGAP/Rac1 axis (Fig. 7). In cultured KINGS‐1 cell line, depletion of endogenous FilGAP by its specific siRNAs increased highly elongated mesenchymal morphology but did not change the total amount of GTP‐Rac1. Therefore, FilGAP may not regulate cell morphology through down‐regulation of total GTP‐Rac1. The mechanism of how FilGAP controls cell morphology is unclear but it is possible that localized inactivation of Rac1 by FilGAP may be important or FilGAP may affect downstream effectors other than Rac1. Further study is required to elucidate the mechanism of how FilGAP regulates glioma cell morphology and migration in vitro and in vivo.

In conclusion, this study clearly provided evidence that FilGAP, as well as IDH1 status, may be useful for predicting the behavior of astrocytomas. In addition, the FilGAP/Rac1 axis may serve as an important regulator of tumor progression in GBMs, probably through alteration of cell morphology.

## Conflict of Interest

Potential conflicts do not exist.

## Supporting information


**Figure S1.** Expression of FilGAP and its related molecules in normal brain. Staining by hematoxylin and eosin (HE) and by IHC for FilGAP, FLNa, integrin *β*2, ECT2, Rac1, and Ki‐67 in normal brain. Astrocytes in closed boxes are magnified in the insets. Note the weak immunoreactivity for FilGAP and FLNa in nerve cells (indicated by arrows). Original magnification, ×100 and ×400 (inset).

**Figure S2.** Expression of FilGAP mRNA expression in astrocytoma. Staining by HE (upper left), IHC (upper right) for FilGAP protein, and ISH (lower) for its mRNA. Note the positive FilGAP mRNA signals (indicated by arrows) in astrocytoma cells, consistent with the strong immunoreactivity. Original magnification, ×200 and ×400 (inset).

**Figure S3.** Expression of Rac1 in astrocytoma cells. (A) Plasma membrane (indicated by long arrows)/perinuclear Rac1 immunoreactivity (indicated by short arrows) in astrocytoma cells with intense cytoplasmic staining. (B) IHC score for cytoplasmic (Cyt) Rac1 in astrocytoma cells with or without plasma membrane/perinuclear (PM/PN) Rac1 staining. The data shown are means±SDs.

**Figure S4.** Relationship of *IDH1* mutations with FilGAP and its related molecules. (A) IHC scores for FilGAP, FLNa, integrin *β*2, and cytoplasmic (Cyt) ECT2 and Rac1, and (B) LIs of nuclear (Nuc) ECT2 and Ki‐67 between wild‐type and mutant (mt) forms of *IDH1* in astrocytomas. The data shown are means±SDs.

**Table S1**. Relationship between gene mutation and protein status of IDH1 gene in astrocytomas.

**Table S2**. Relationship of plasma membrane/perinuclear Rac 1 expression with tumor grade and in astrocytomas.

**Table S3**. Correlations among expression of FilGAP and its related molecules in tumor core and periphery of GBMs.
