# Supplementary figures and images for: The role of FilGAP, a Rac‐specific Rho‐GTPase‐activating protein, in tumor progression and behavior of astrocytomas
Source: Cancer Med. 2016 Oct 27;5(12):3412–25. doi: 10.1002/cam4.937 (PMC5224849; doi:10.1002/cam4.937)

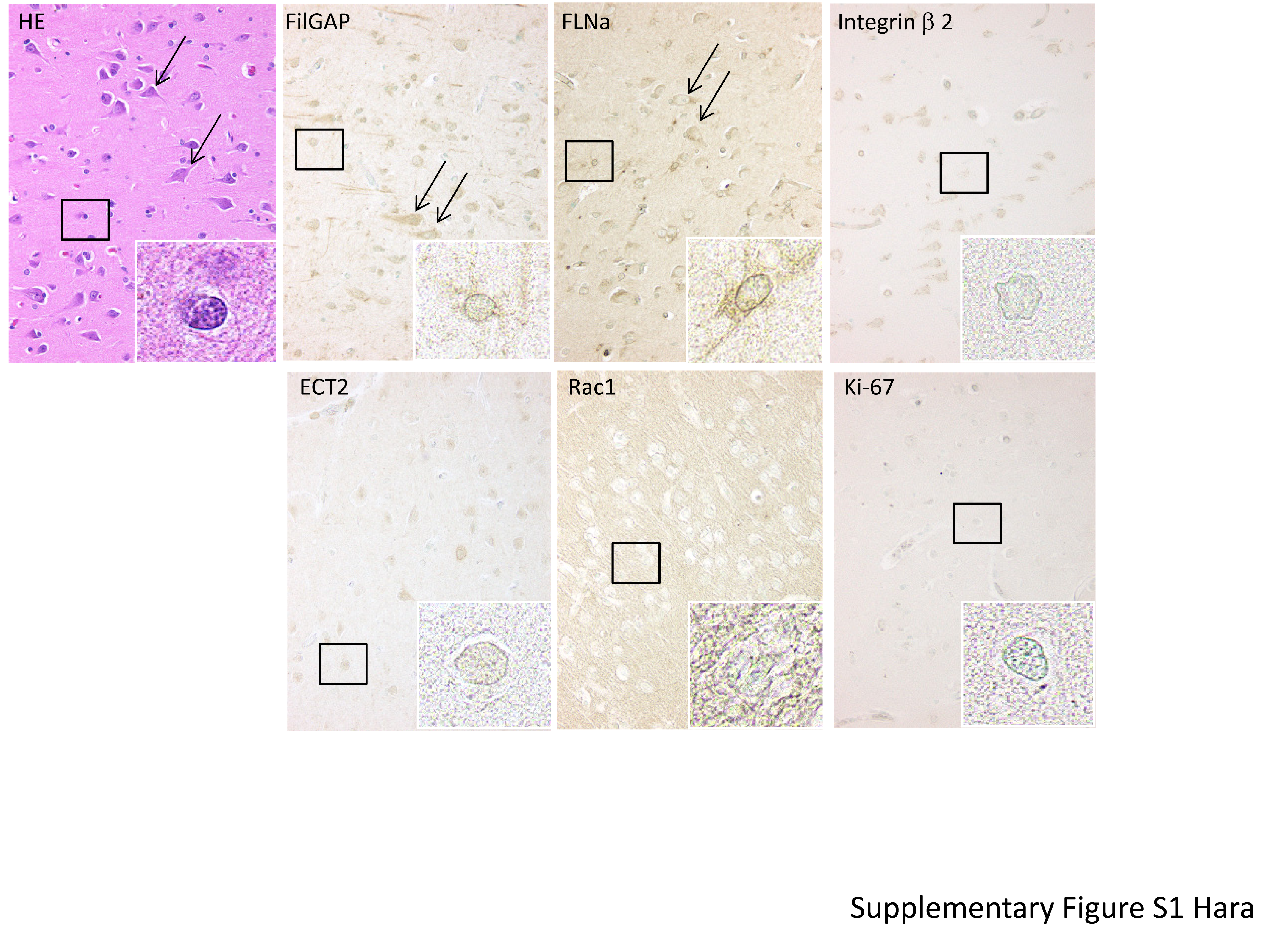

Supplement: Supplementary file 1 — Figure S1. Expression of FilGAP and its related molecules in normal brain. Staining by hematoxylin and eosin (HE) and by IHC for FilGAP, FLNa, integrin β2, ECT2, Rac1, and Ki‐67 in normal brain. Astrocytes in closed boxes are magnified in the insets. Note the weak immunoreactivity for FilGAP and FLNa in nerve cells (indicated by arrows). Original magnification, ×100 and ×400 (inset). [file CAM4-5-3412-s001.tif]

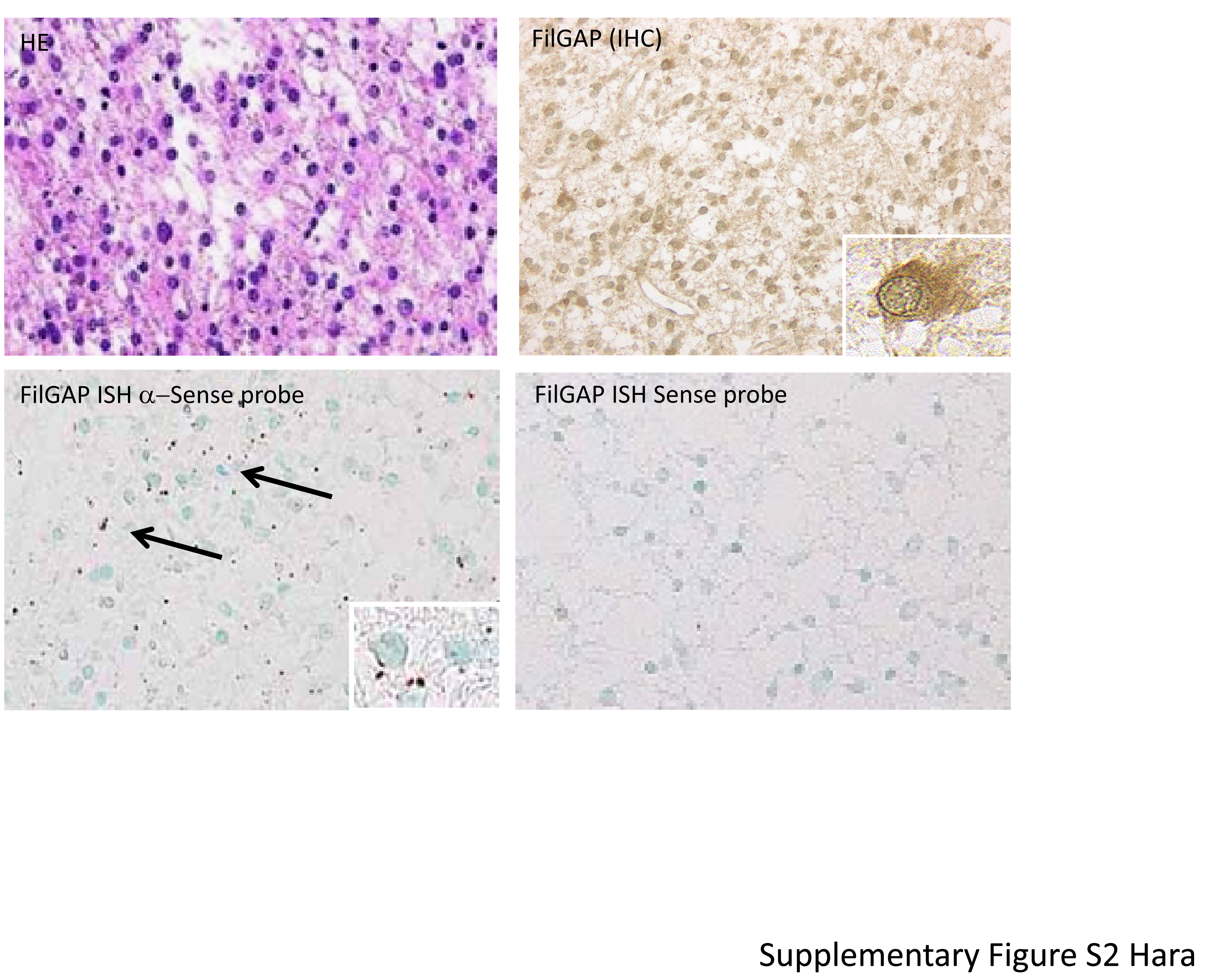

Supplement: Supplementary file 2 — Figure S2. Expression of FilGAP mRNA expression in astrocytoma. Staining by HE (upper left), IHC (upper right) for FilGAP protein, and ISH (lower) for its mRNA. Note the positive FilGAP mRNA signals (indicated by arrows) in astrocytoma cells, consistent with the strong immunoreactivity. Original magnification, ×200 and ×400 (inset). [file CAM4-5-3412-s002.tif]

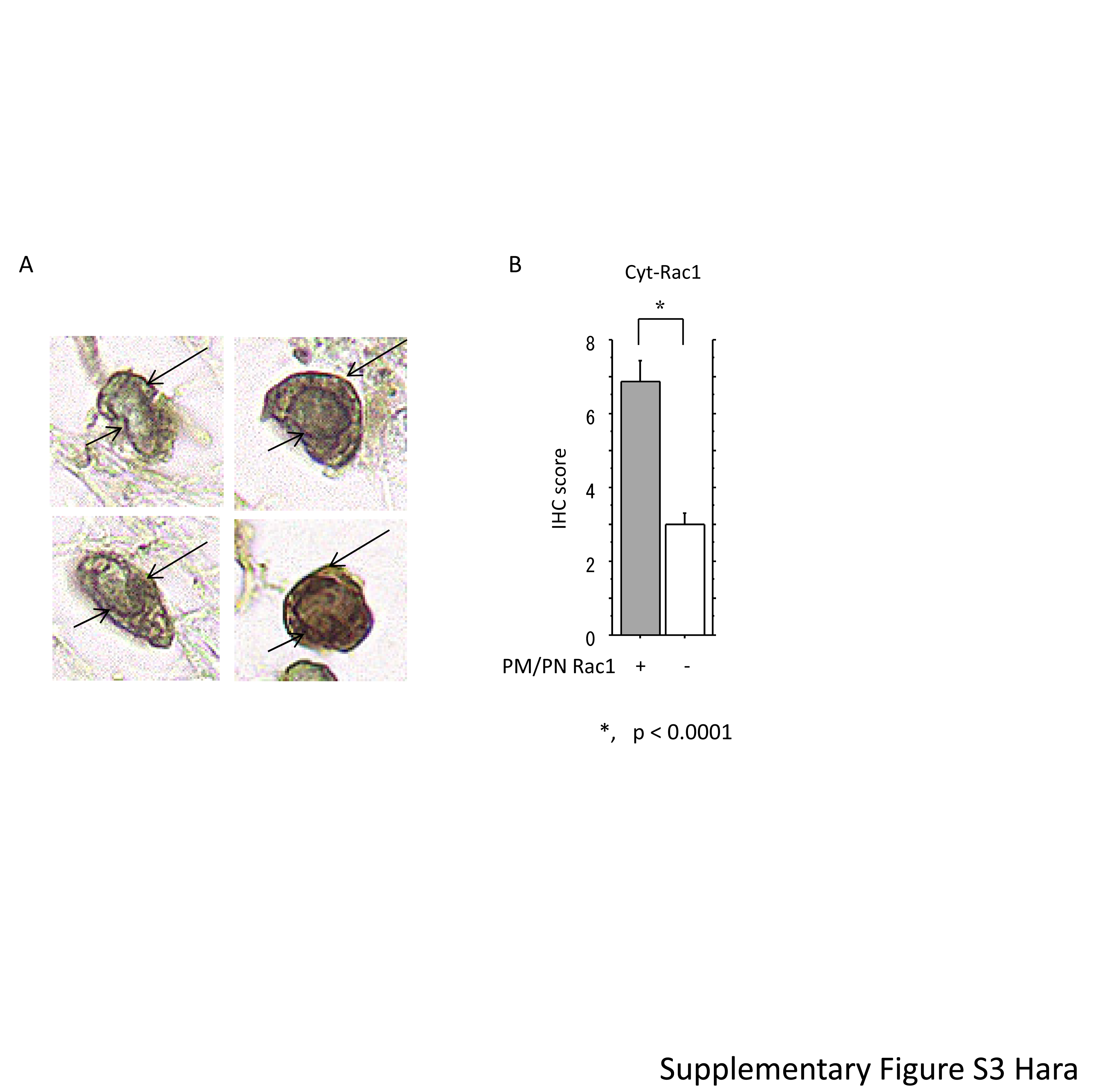

Supplement: Supplementary file 3 — Figure S3. Expression of Rac1 in astrocytoma cells. (A) Plasma membrane (indicated by long arrows)/perinuclear Rac1 immunoreactivity (indicated by short arrows) in astrocytoma cells with intense cytoplasmic staining. (B) IHC score for cytoplasmic (Cyt) Rac1 in astrocytoma cells with or without plasma membrane/perinuclear (PM/PN) Rac1 staining. The data shown are means±SDs. [file CAM4-5-3412-s003.tif]

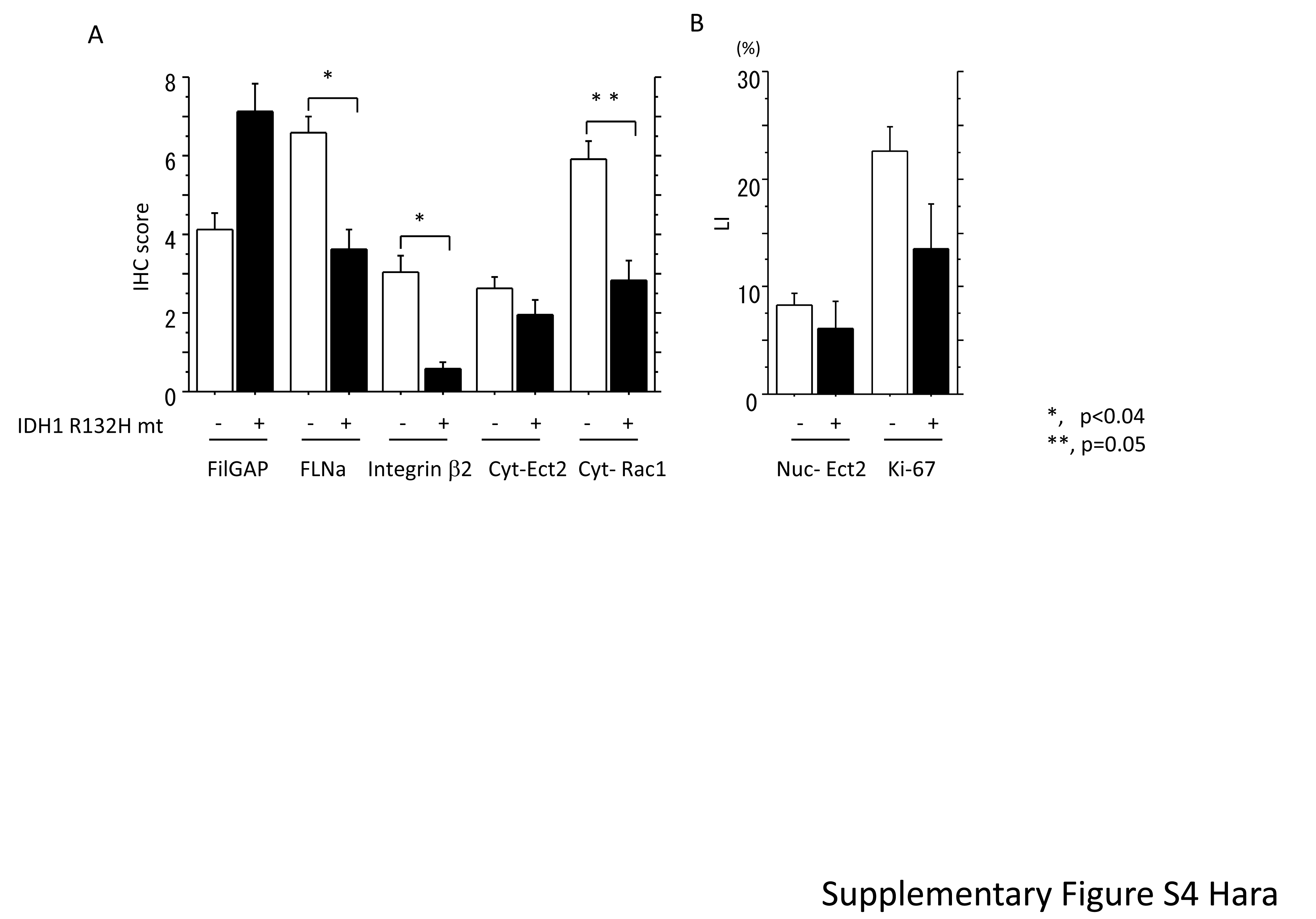

Supplement: Supplementary file 4 — Figure S4. Relationship of IDH1 mutations with FilGAP and its related molecules. (A) IHC scores for FilGAP, FLNa, integrin β2, and cytoplasmic (Cyt) ECT2 and Rac1, and (B) LIs of nuclear (Nuc) ECT2 and Ki‐67 between wild‐type and mutant (mt) forms of IDH1 in astrocytomas. The data shown are means±SDs. [file CAM4-5-3412-s004.tif]
