# Supplementary material for: The role of FilGAP, a Rac‐specific Rho‐GTPase‐activating protein, in tumor progression and behavior of astrocytomas
Source: Cancer Med. 2016 Oct 27;5(12):3412–25. doi: 10.1002/cam4.937 (PMC5224849; doi:10.1002/cam4.937)
Supplement: Supplementary file 2 [file CAM4-5-3412-s005.docx]

Supplementary Table S1 Relationship between gene mutation and protein status of *IDH1* gene in astrocytomas

		Gene status		p-value
		Wild<br>n (%)	Mutant<br>n (%)	
Protein status (IHC)	Positive	16 (14.5)	22 (20)	<0.0001
	Negative	71 (64.5)	1 (0.9)	

IHC, immunohistochemistry; n, number of cases
