# Supplementary material for: The role of FilGAP, a Rac‐specific Rho‐GTPase‐activating protein, in tumor progression and behavior of astrocytomas
Source: Cancer Med. 2016 Oct 27;5(12):3412–25. doi: 10.1002/cam4.937 (PMC5224849; doi:10.1002/cam4.937)
Supplement: Supplementary file 3 [file CAM4-5-3412-s006.docx]

Supplementary Table S2 Relationship of plasma membrane/perinuclear Rac1 expression with tumor grade and in astrocytomas

		PM/PN Rac1 expression		
		Positive n (%)	Negative n (%)	p-value
Tumor grade	Grade II	8 (16.7)	40 (83.3)	0.0009
	Grade III	9 (31)	20 (69)	
	Grade IV	25 (52)	23 (48)	
Tumor lesion (Grade IV)	Core	27 (73)	10 (27)	<0.0001
	Peripheral	5 (13.5)	32 (86.5)	

PM/PN, plasma membrane/perinuclear; n, number of cases
