# Supplementary material for: The role of FilGAP, a Rac‐specific Rho‐GTPase‐activating protein, in tumor progression and behavior of astrocytomas
Source: Cancer Med. 2016 Oct 27;5(12):3412–25. doi: 10.1002/cam4.937 (PMC5224849; doi:10.1002/cam4.937)
Supplement: Supplementary file 4 [file CAM4-5-3412-s007.docx]

Supplementary Table S3 Correlations among expression of FilGAP and its related molecules in tumor core and periphery of GBMs

	FilGAP ρ (p)	Filamin A ρ (p)	Integrin β2 ρ (p)	Cyto ECT2 ρ (p)	Nuc ECT2 ρ (p)	Cyto Rac1 ρ (p)
Filamin A	-0.41 (0.0003)	*	*	*	*	*
Integrin β2	-0.36 (0.002)	0.17 -0.15	*	*	*	*
Cyto ECT2	-0.51 (<0.0001)	0.72 (<0.0001)	0.39 (0.0006)	*	*	*
Nuc ECT2	-0.1 (<0.0001)	0.14 -0.28	0.17 (0.18)	0.33 (0.01)	*	*
Cyto Rac1	-0.55 (<0.0001)	0.64 (<0.0001)	0.4 -0.0004	0.76 (<0.0001)	0.25 -0.05	*
Ki-67	-0.55 (<0.0001)	0.51 (0.003)	0.1 (0.4)	0.44 (0.0002)	0.63 (<0.0001)	0.63 (<0.0001)

ρ, Sperman's correlation coefficient; *, not examined; Cyto, cytoplasmic; Nuc, nuclear; GBM, glioblastoma
